# *Drosophila* as a host model to investigate toll-mediated innate immune evasion of human pathogenic fungi

**DOI:** 10.3389/fcimb.2026.1853190

**Published:** 2026-07-17

**Authors:** Widya Hardiyanti, Muhammad Rasul Pratama, Emil Salim, Firzan Nainu

**Affiliations:** 1 Study Program of Pharmacy, Faculty of Medicine and Health Sciences, Universitas Muhammadiyah Makassar, Makassar, Indonesia; 2 Unhas Fly Research Group, Faculty of Pharmacy, Hasanuddin University, Makassar, Indonesia; 3 Study Program of Pharmacy, Stikes Bhakti Pertiwi Luwu Raya Palopo, Palopo, Indonesia; 4 Department of Pharmacology and Clinical/Community Pharmacy, Faculty of Pharmacy, Universitas Sumatera Utara, Medan, Indonesia; 5 Department of Pharmacy, Faculty of Pharmacy, Hasanuddin University, Makassar, Indonesia

**Keywords:** fruit flies, fungal infections, immune evasion, innate immunity, toll pathway

## Introduction

Fungal infections represent a substantial and increasing global health burden. Annually, more than 6.5 million individuals develop invasive fungal diseases, leading to approximately 3.8 million deaths, of which around 2.5 million are directly attributable to fungal pathogens (Denning, 2024). In response, the World Health Organization has established the Fungal Priority Pathogens List to guide research and public health strategies by identifying high-impact pathogens with significant unmet needs (WHO, 2022). A major contributor to fungal pathogenicity is the ability of these organisms to evade host immunity through mechanisms that impair immune recognition, suppress defense signaling, and modulate inflammatory responses. Despite their importance, studying fungal immune evasion in mammalian systems remains challenging. These models are associated with high costs, ethical constraints, and regulatory limitations, which restrict experimental scalability and flexibility (Lionakis et al., 2023). In addition, mammalian models are inherently low-throughput due to long generation times, complex physiology, and labor-intensive methodologies, thereby limiting large-scale genetic and pharmacological investigations (Kiani et al., 2022).

*Drosophila melanogaster* has emerged as a valuable *in vivo* model for studying host–fungal interactions due to its conserved innate immune pathways and strong genetic tractability. Approximately 75% of human disease-associated genes possess homologs in the fly genome, while antifungal Toll/NF-κB signaling exhibits significant functional conservation with mammalian innate immune responses (Avila et al., 2024). In addition to practical advantages such as low maintenance cost, rapid generation time, and high reproductive capacity, *D. melanogaster* is highly amenable to high-throughput infection and genetic screening approaches (Younes et al., 2020). The availability of sophisticated genetic tools, including RNA interference, CRISPR-based genome engineering, and tissue-specific expression systems, further enables precise dissection of host–pathogen interactions at cellular and molecular levels (Port et al., 2020). Collectively, these attributes establish *D. melanogaster* as a powerful complementary model for elucidating fungal immune evasion strategies, including modulation of pattern recognition receptors (PRRs), masking of pathogen-associated molecular patterns (PAMPs), and suppression of antifungal immune responses, with particular emphasis on Toll-mediated antifungal signaling and its downstream effectors, while additional defense mechanisms such as melanization, hemocyte-mediated cellular immunity, JAK/STAT signaling, and epithelial barrier responses are addressed only briefly.

## Innate immune evasion of human pathogenic fungi: a critical knowledge gap

Human pathogenic fungi employ diverse strategies to evade innate immune recognition and clearance, posing a major challenge to understanding fungal pathogenesis. *Candida albicans* dynamically modulates β-1,3-glucan exposure in response to host-like cues such as CO_2_, hypoxia, and lactate, while secreted glucanases remodel the cell wall during colonization and infection, restricting β-1,3-glucan exposure to discrete foci and profoundly altering Dectin-1-mediated recognition (de Assis et al., 2022; Avelar et al., 2024). *Cryptococcus neoformans* utilizes a glucuronoxylomannan-rich polysaccharide capsule, Titan cell formation, and melanin production to impair phagocytosis and modulate cytokine and complement responses, thereby promoting persistence and dissemination (Li et al., 2025). Similarly, *Aspergillus fumigatus* produces melanin to protect conidia from oxidative stress and secretes virulence factors such as gliotoxin that inhibit phagocytic activity. Collectively, these fungi share conserved immune evasion mechanisms, including Pathogen-Associated Molecular Patterns masking, immunomodulation, metabolic adaptation, and intracellular survival. Among these, β-1,3-glucan masking is particularly well-characterized in *C. albicans* (Chen et al., 2022; Earle et al., 2023; Ma et al., 2024).

Comparable immune evasion mechanisms have been characterized using *D. melanogaster* as a model system. Entomopathogenic fungi such as *Metarhizium robertsii* secrete the effector Fkp1, which binds the hemolymph cathepsinK1 (CtsK1) and prevents activation of the danger-sensing protease persephone, thereby suppressing Toll signaling and antifungal gene expression (Tang et al., 2025). In *Drosophila*, fungal infection is sensed through two complementary pathways: β-glucan recognition mediated by Gram-Negative Binding Protein 3 (GNBP3) and protease-activated persephone pathway (Lu et al., 2024; Tang et al., 2025). These dual systems illustrate how fungal pathogens can disrupt both pattern recognition and danger signaling to evade host immunity. Additionally, pathogens such as Entomophthora muscae adopt modified or protoplastic growth forms that reduce exposure of immunogenic cell wall components, enabling persistence despite intact Toll-mediated responses (Liu et al., 2026).

**Figure 1:**
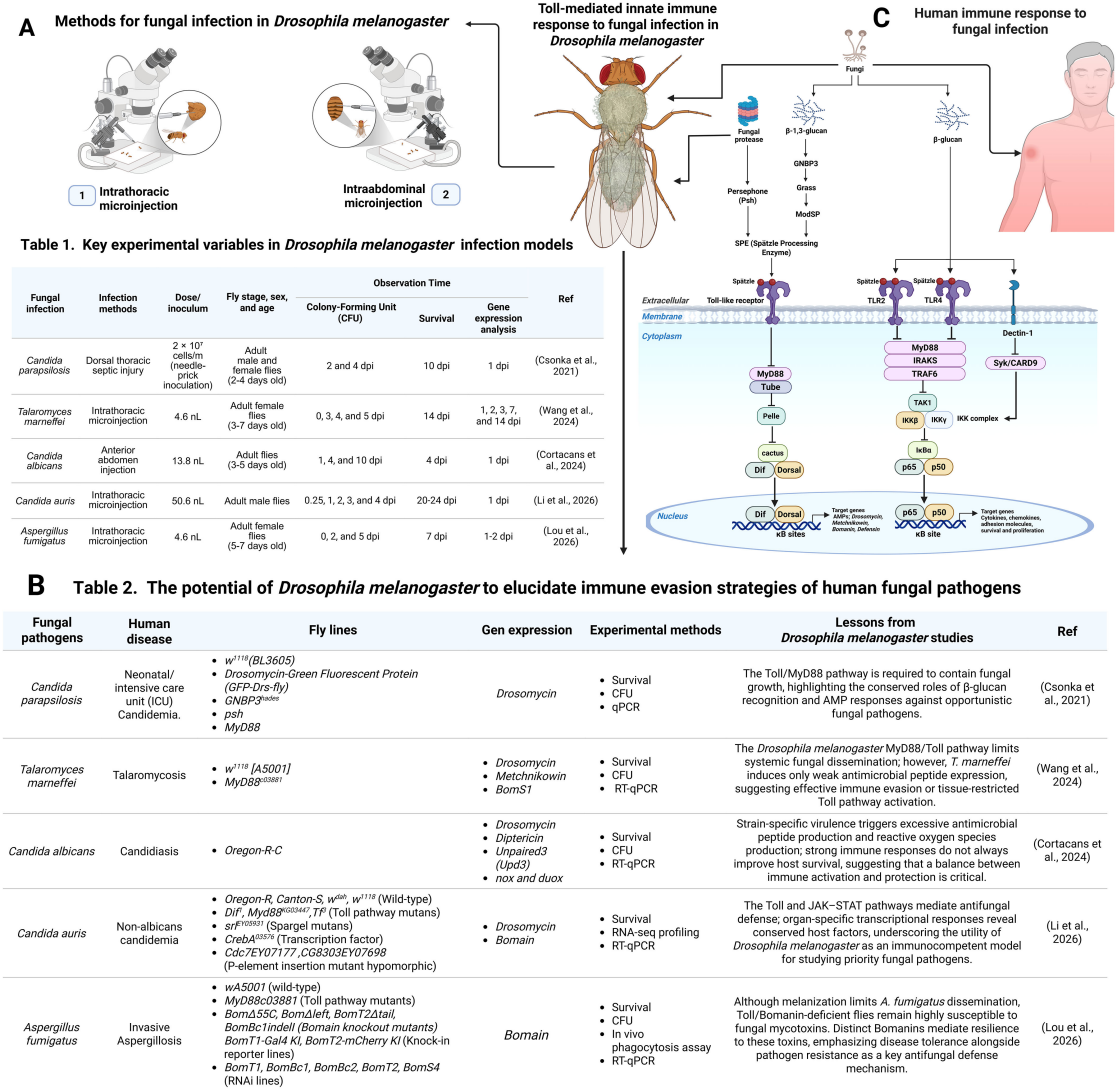
*Drosophila melanogaster* as a model for investigating fungal pathogenesis, host–pathogen interactions, and Toll-mediated antifungal immunity. **(A)** Experimental fungal infection approaches in *D. melanogaster*, including intrathoracic microinjection and abdominal inoculation, key infection parameters used for major human fungal pathogens, and the application of *D. melanogaster* genetic models to dissect fungal virulence, immune-evasion mechanisms, and host defense pathways. **(B)** Mechanisms of fungal immune evasion and host recognition in *D. melanogaster*, including β-glucan recognition by Gram-Negative Binding Protein 3 (GNBP3), activation of the Persephone-mediated danger-sensing pathway, fungal interference with Toll signaling, and evasion of Toll-regulated antifungal effectors. **(C)** Comparative overview of conserved antifungal immune signaling pathways in *D. melanogaster* and humans, including fungal recognition, Toll/Toll-like receptor signaling, downstream signal transduction, and induction of antimicrobial peptides (AMPs) and nuclear factor kappa B (NF-κB)-dependent immune responses. These conserved mechanisms highlight the utility of *D. melanogaster* as a powerful *in vivo* model for elucidating fungal pathogenesis, antifungal immunity, immune evasion, and disease-tolerance mechanisms relevant to human mycoses. AMP, antimicrobial peptide; dpi, days post-infection; GNBP3, Gram-Negative Binding Protein 3; IKK, inhibitor of nuclear factor kappa-B kinase; IKKα, IκB Kinase Alpha; IKKβ, IκB Kinase Beta; IKKγ, IκB Kinase Gamma; IRAKs, Interleukin-1 Receptor-Associated Kinases; IκBα, Inhibitor of Nuclear Factor Kappa B Alpha; TAK1, Transforming Growth Factor-β Activated Kinase 1; JAK–STAT, Janus kinase–signal transducer and activator of transcription; ModSP, modular serine protease; MyD88, myeloid differentiation primary response protein 88; NF-κB, nuclear factor kappa B; Syk, spleen tyrosine kinase; TLR2, Toll-like receptor 2; TLR4, Toll-like receptor 4; TRAF6, tumor necrosis factor receptor-associated factor 6; p56 RelA (NF-κB subunit p65); p50, RelA (NF-κB1 p50 subunit). Created in BioRender. Nainu, F. (2026) https://BioRender.com/0x7qv3e.

Fungal infection in *D. melanogaster* is commonly induced by septic injury or microinjection (Rai et al., 2023; Yuvaraj et al., 2026) (**Figure 1A**), enabling precise delivery of fungal cells into the hemocoel and controlled study of systemic Toll-mediated immune responses (Liu et al., 2026). Although these methods bypass natural barriers, oral and environmental exposure models better represent early fungal-epithelial interactions in gut and respiratory tissues. This genetically tractable model supports detailed analysis of host–fungal interactions, including mechanisms that disrupt β-glucan recognition, inhibit persephone-dependent Toll activation, and evade Toll-regulated effectors such as Bomanins (Cohen et al., 2020; Tay and Hsueh, 2025) (**Figure 1B**). While the absence of adaptive immunity allows focused investigation of innate responses and high-throughput studies, it limits modeling of complex vertebrate host–fungal interactions (Mpamhanga and Kounatidis, 2024).

## *Drosophila melanogaster* activates innate immune pathways in response to fungal infection

*D. melanogaster* mounts innate immune responses to fungal infection through multiple pathways, but Toll signaling represents the best-characterized antifungal axis and therefore constitutes the primary antifungal defense pathway (Mpamhanga and Kounatidis, 2024). Recognition is initiated when β-1,3-glucan, a major component of the fungal cell wall, is detected by the Pattern Recognition Receptor (PRR) GNBP3 in the hemolymph (Yu et al., 2022; Mpamhanga and Kounatidis, 2024). Binding of β-glucan to GNBP3 triggers an extracellular serine protease cascade involving Modular Serine Protease (ModSP), Grass, and the Spätzle processing enzyme (SPE), ultimately resulting in proteolytic activation of the cytokine-like ligand Spätzle (Shan et al., 2023; Tang et al., 2025). Cleaved Spätzle binds to Toll-like receptors on fat body cells and hemocytes, initiating intracellular signaling that culminates in antimicrobial peptide gene expression (Shan et al., 2023; Mpamhanga and Kounatidis, 2024). In addition to this canonical PRR-mediated pathway, secreted fungal proteases can directly activate the hemolymph serine protease persephone, which then engages the downstream serine protease cascade and promotes activation of the Spätzle-processing enzyme. This protease-triggered ‘danger’ pathway can sustain Toll signaling independently of GNBP3−mediated recognition and both the PRR cascade (GNBP3→ModSP→Grass→SPE) and the persephone branch ultimately lead to proteolytic activation of Spätzle (Liu et al., 2026). This convergence on Spätzle activates the Toll-like receptors, leading to intracellular signaling that results in Cactus degradation and nuclear translocation of the NF-κB homologs Dif and Dorsal, thereby inducing transcription of antifungal antimicrobial peptide genes such as *drosomycin* and *metchnikowin* (Chen et al., 2025).

Human innate immune recognition of fungi is mediated by multiple PRRs. Among these, Dectin-1-mediated recognizes β(1,3)-glucans in the fungal cell wall, while Toll-like receptors (TLRs), particularly Toll-like Receptors 2 (TLR2) and TLR4, cooperate in detecting fungal cell wall glycoconjugates; notably, TLR4 recognizes mannans and related mannose-containing polysaccharides (Sohail Sajid et al., 2022; Kozłowska et al., 2025). In *D. melanogaster*, fungal β-glucans are recognized primarily by GNBP3 and via activation of the persephone-mediated danger-sensing pathway (Lu et al., 2024; Mpamhanga and Kounatidis, 2024; Tang et al., 2025). Although these receptors are not direct orthologs, they function as analogous systems for fungal sensing and initiation of antifungal immunity. In both mammals and *D. melanogaster*, fungal recognition ultimately converges on NF-κB-related signaling pathways. In mammals, Dectin-1-mediated recognition activates Syk/CARD9 signaling, whereas TLR signaling proceeds through Myeloid Differentiation Primary Response Protein 88 *MyD88*-dependent pathways to induce inflammatory responses (Mata-Martínez et al., 2022; Chen et al., 2025). Similarly, in Drosophila, Toll pathway activation drives nuclear translocation of the NF-κB-related transcription factors Dif and Dorsal, which induce antifungal antimicrobial peptide genes (Brutscher and Basler, 2025). Thus, the most striking conservation lies in the overall architecture of PRR-triggered NF-κB-dependent innate immune signaling cascades, rather than one-to-one conservation of receptors or effector repertoires (Chen et al., 2025). A schematic comparison of antifungal immune responses in *D. melanogaster* and humans is presented in **Figure 1C**.

## Potential of *D. melanogaster* to elucidate innate immune evasion strategies of human fungal pathogens

*D. melanogaster* offers a powerful platform to dissect how human fungal pathogens evade innate immunity. This is largely due to the genetic tractability of key immune components, including Toll/*MyD88* signaling, hemocyte-mediated phagocytosis, and antimicrobial peptide (AMP) effectors, which can be systematically manipulated while monitoring host survival, fungal burden, and tissue-specific responses (Csonka et al., 2021; Wang et al., 2024). Studies using *C. albicans* and *C. parapsilosis* show that conserved β-glucan sensing through the Toll/*MyD88* axis is essential to limit fungal growth and that highly virulent strains provoke excessive AMP and Reactive Oxygen Species (ROS) induction, indicating that immune over-activation can be as detrimental as insufficient responses, a key concept for understanding inflammatory pathology in candidemia (Csonka et al., 2021; Cortacans et al., 2024). Similarly, investigations of *Candida auris* infection show that antifungal defense depends on coordinated Toll and JAK/STAT signaling, while organ-specific transcriptional responses identify conserved host factors that may contribute to fungal pathogenesis across species, highlighting the translational relevance of the *D. melanogaster* model (Li et al., 2026). In the context of *A. fumigatus* infection, studies in *D. melanogaster* have further revealed that Toll signaling promotes host resilience not only by restricting fungal invasion but also by protecting against the effects of secreted mycotoxins through specific short-form Bomanins, thereby highlighting the distinction between resistance and disease-tolerance mechanisms that are highly relevant to invasive aspergillosis (Huang et al., 2023; Xu et al., 2023). Finally, infection with *Talaromyces marneffei* elicits an atypical *MyD88*/Toll-dependent response in which systemic AMP induction remains limited despite strong dependence on *MyD88* signaling, suggesting either localized Toll activation or partial immune evasion and providing a tractable system to investigate immune evasion strategies that may parallel those in talaromycosis patients (Wang et al., 2024).

## Discussion

Antifungal defense in Drosophila is mediated by coordinated innate immune mechanisms, including Toll-dependent NF-κB signaling, melanization cascades, hemocyte-mediated phagocytosis, epithelial barrier immunity, and JAK/STAT signaling, which collectively parallel key features of vertebrate antifungal responses. Fungal recognition occurs through β-glucan-binding pattern recognition receptors and a protease-mediated danger-sensing cascade, leading to activation of the NF-κB homologs Dif and Dorsal (Mpamhanga and Kounatidis, 2024; Liu et al., 2026). This activation drives the expression of antifungal effectors, including antimicrobial peptides such as *Drosomycin* and *Bomanins* (Mpamhanga and Kounatidis, 2024). Together, these integrated responses function to restrict fungal proliferation and limit systemic dissemination within the host.

Accumulating evidence indicates that fungal pathogens can evade or reprogram immune responses in *D. melanogaster*, providing a tractable framework for studying conserved antifungal immune evasion strategies. Entomopathogenic fungi such as *Metarhizium* and *Beauveria* secrete the effector Fkp1, which inhibits activation of the danger-sensing protease persephone, thereby suppressing Toll signaling and AMP production (Tang et al., 2025; Tay and Hsueh, 2025). Recent mechanistic studies further show that Toll-regulated Bomanins promote resilience by neutralizing *Aspergillus mycotoxins*, and that Toll-1 activation in the nervous system can drive Sarm- and JNK-dependent neural cell death instead of antimicrobial peptide induction, providing tractable models of toxin-mediated tolerance and tissue-specific immune pathology (Xu et al., 2023; Singh et al., 2025; Lou et al., 2026). Infection with *Talaromyces marneffei*, which elicits a *MyD88*/Toll-dependent response with limited systemic AMP upregulation, suggests localized or partially evasive Toll signaling and illustrates how fungal pathogens differentially shape Toll outputs across tissues and disease contexts (Mpamhanga and Kounatidis, 2024; Wang et al., 2024). These mechanisms parallel mammalian fungal immune evasion strategies, including β-glucan masking, suppression of NF-κB signaling, and impaired phagocyte function (Sohail Sajid et al., 2022; Earle et al., 2023; Li et al., 2025).

Despite recent advances, a key knowledge gap remains in understanding how fungal pathogens selectively target different components of innate immune signaling to balance immune evasion with host viability. Addressing this question in mammalian systems is challenging because of their complexity. By contrast, *D. melanogaster* enables systematic dissection of immune evasion mechanisms across multiple layers of host defense in a whole-organism context. Notably, fungal interference with β-glucan recognition in flies parallels evasion of Dectin-1 signaling in mammals, and disruption of Toll-mediated NF-κB activation reflects analogous targeting of TLR–*MyD88* pathways, highlighting conserved principles of innate immune evasion; however, the absence of adaptive immunity, specialized myeloid subsets, and complex tissue microenvironments in *Drosophila* limits direct extrapolation of these findings to the full spectrum of mammalian host–fungal interactions.

*D. melanogaster* provides a highly tractable system for studying antifungal immunity, supported by genetic tools such as RNAi, CRISPR-based genome editing, and tissue-specific transgenesis (Victor Atoki et al., 2025). These enable precise manipulation of immune pathways alongside rapid, quantifiable readouts including survival, fungal burden, AMP expression, melanization, and phagocytosis. Systemic infection models using hemocoelic inoculation are commonly used to investigate Toll-centered antifungal signaling. However, these approaches bypass natural barrier defenses (Liu et al., 2026). Barrier-associated immunity, including epithelial responses, ROS production, and mucosal host–fungal interactions, represents a complementary aspect that may involve distinct mechanisms. Therefore, findings from systemic models should be interpreted cautiously, particularly for mucosal fungal infections in humans (Iliev et al., 2025). *D. melanogaster* has important limitations as a model for fungal infection, including the absence of adaptive immunity, specialized myeloid cell subsets, and complex tissue environments found in humans. Its use is therefore best suited to studying innate immune processes, particularly Toll-mediated antifungal responses, rather than providing a comprehensive view of all immune pathways. Despite these constraints, it serves as a valuable complementary platform for identifying conserved immune evasion mechanisms. Recent studies integrating genetic, transcriptomic, and imaging approaches have significantly advanced understanding of *D. melanogaster* antifungal defenses. Comparative analyses with mammalian systems will further clarify conserved strategies and support the development of antifungal therapies.
